# Oral bacteria colonize and compete with gut microbiota in gnotobiotic mice

**DOI:** 10.1038/s41368-018-0043-9

**Published:** 2019-03-05

**Authors:** Bolei Li, Yang Ge, Lei Cheng, Benhua Zeng, Jinzhao Yu, Xian Peng, Jianhua Zhao, Wenxia Li, Biao Ren, Mingyun Li, Hong Wei, Xuedong Zhou

**Affiliations:** 1 State Key Laboratory of Oral Diseases & National Clinical Research Center for Oral Diseases, Sichuan University, Chengdu, China; 2 Department of Cariology and Endodontics, West China Hospital of Stomatology, Sichuan University, Chengdu, China; 3 Department of Laboratory Animal Science, College of Basic Medical Sciences, Third Military Medical University, Chongqing, China; 4 Shanghai Majorbio Bio-pharm Technology Co., Ltd, Shanghai, China

**Keywords:** Microbial ecology, Microbiome, Microbial ecology, Microbiome

## Abstract

The oral microbiota is associated with oral diseases and digestive systemic diseases. Nevertheless, the causal relationship between them has not been completely elucidated, and colonisation of the gut by oral bacteria is not clear due to the limitations of existing research models. The aim of this study was to develop a human oral microbiota-associated (HOMA) mouse model and to investigate the ecological invasion into the gut. By transplanting human saliva into germ-free (GF) mice, a HOMA mouse model was first constructed. 16S rRNA gene sequencing was used to reveal the biogeography of oral bacteria along the cephalocaudal axis of the digestive tract. In the HOMA mice, 84.78% of the detected genus-level taxa were specific to the donor. Principal component analysis (PCA) revealed that the donor oral microbiota clustered with those of the HOMA mice and were distinct from those of specific pathogen-free (SPF) mice. In HOMA mice, OTU counts decreased from the stomach and small intestine to the distal gut. The distal gut was dominated by *Streptococcus, Veillonella, Haemophilus, Fusobacterium, Trichococcus* and *Actinomyces*. HOMA mice and human microbiota-associated (HMA) mice along with the GF mice were then cohoused. Microbial communities of cohoused mice clustered together and were significantly separated from those of HOMA mice and HMA mice. The Source Tracker analysis and network analysis revealed more significant ecological invasion from oral bacteria in the small intestines, compared to the distal gut, of cohoused mice. In conclusion, a HOMA mouse model was successfully established. By overcoming the physical and microbial barrier, oral bacteria colonised the gut and profiled the gut microbiota, especially in the small intestine.

## A mouse model for human oral bacteria studies

Swabbing the mouths of germ-free mice with human saliva can provide an effective mouse model for studying the roles of human oral bacteria in mouth and gut diseases. The human oral microbiota-associated (HOMA) mice were developed by Lei Cheng of Sichuan University and colleagues in China and bred alone for 35 days. A significant proportion (85%) of the types of bacteria present in the human donors’ saliva successfully established themselves in the germ-free mice and colonised various parts of their guts. When the HOMA mice were co-bred with other types of mouse models, digestive tract bacteria transferred to them, preventing oral bacteria from significantly colonising the gut. The researchers conclude that a HOMA model was successfully established and could help overcome current limitations in investigations related to human oral bacteria and disease.

## Introduction

Clinical trials have indicated that the oral microbiota is associated with dental caries and periodontitis,^1–4^ both of which give rise to an extensive loss of natural teeth in older people and are identified as public health problems worldwide.^5^ Accumulating evidence has even linked the human oral microbiota to oral cancer.^6,7^ In recent years, oral microecology dysbiosis has been proven to cause periodontitis^4,8^ and regarded as an indicator to predict early childhood caries (ECC).^9^ Thus, the oral microbiota has a key role in the initiation of oral diseases.

An increasing number of clinical research studies of the oral microbiota are being designed. However, the clinical investigations are usually restricted by complex conditions, including ethical issues. Regardless, a prospective cohort clinical study^9^ found that shifts in the microbiota preceded the manifestation of clinical symptoms of ECC. Unfortunately, most of the other studies were cross-sectional and could barely address whether the oral microbiota was the cause or effect in the development of oral diseases. In-vitro models also have limitations due to the abundant uncultivated phylotypes in the mouth.^10^ Animal models would have been considered a good choice to study the oral microbiota; however, the oral microbiota of mice, the most common experiment animal model, differs from that of humans. Therefore, a HOMA mouse model, with an oral microbiota similar to the human donors, must be established to reveal the cause-and-effect relationships between the oral microbiota and host pathologies, like the HMA mouse model.^11,12^

Not only oral diseases but also oral bacteria are linked to various digestive systemic diseases, including inflammatory bowel disease,^13,14^ colorectal cancer (CRC),^15^ pancreatic cancer,^16,17^ liver carcinoma^18^ and liver cirrhosis.^19^ Seedorf et al.^20^ demonstrated that mouth-derived bacteria such as *Actinobacteria, Bacilli, Clostridia, Fusobacteria* and *Epsilonproteobacteria* are able to overcome the host physical barrier and persist in the germ-free distal gut. Comparing the gut microbiome of patients suffering from liver cirrhosis with that of healthy control individuals, Qin et al.^21^ found that most (54%) of the patient-enriched faecal microbial species originated from the oral cavity, demonstrating that the oral microbiota had invaded the gut of patients with liver cirrhosis. These studies indicated that the oral microbiota influenced host health by invading and colonising the gut. The colonisation of oral microbiota in the gut is a key point to understand pathologic colonisation, facilitating studies of the pathogenic mechanisms of oral bacteria in systemic digestive diseases. However, invasion by oral microbiota by overcoming host physical barriers and gut microbiota barriers at various regions along the cephalocaudal axis of the gut is not well described.

**Fig. 1:**
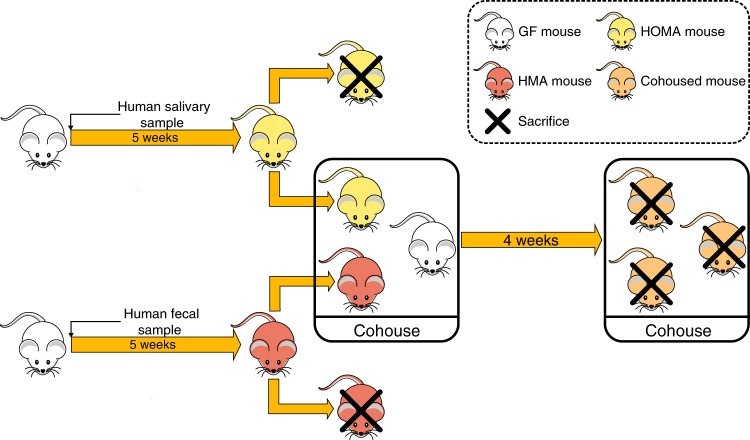
Design of the human microbiota transplant and cohousing experiments

To develop the HOMA mouse model, we introduced the human salivary microbiota into GF mice and created a well-defined, representative animal model of the human oral microbial ecosystem. Using the HOMA mouse model, we investigated the colonisation of gut-selected oral bacteria along the longitudinal axis. Furthermore, we studied the competition of oral microbiota with the native gut microbiota in various regions of the gut and identified key bacteria during the ecological invasion, by cohousing HOMA mice, HMA mice and GF mice (Fig. 1).

## Results

### The oral microbiota of the HOMA mouse model

The surveys of oral samples revealed the engraftment of the human oral microbiota: all bacterial phyla, classes, orders, 27 of 28 bacterial families, and 84.78% (39 of 46) of genus-level taxa were detected among the recipient mice. All seven genus-level taxa missed by the humanised mice exhibited a low abundance in the donor sample (0.21% on average). The oral microbiota of the donor was dominated by eleven genus-level taxa, with a high relative abundance (>1%), of which five, *Veillonella, Fusobacterium, Streptococcus, Porphyromonas* and *Haemophilus*, maintained a high abundance (>1% on average) among the recipient mice. The others were depleted to a low abundance among the recipient mice (Table S1).

**Fig. 2:**
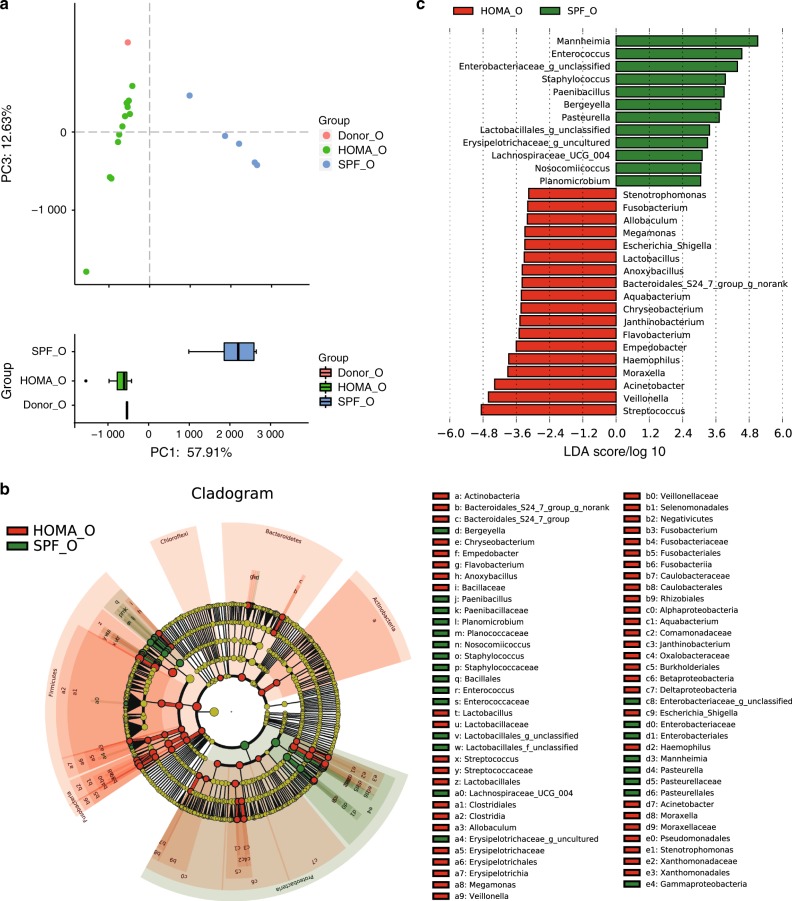
Advancement of the HOMA mouse model. **a** PCA score plot of the oral microbiota of the human donor (Donor_O, red), HOMA mice (HOMA_O, green) and SPF mice (SPF_O, blue) at the genus level. **b** Taxonomic cladogram for HOMA mouse-enriched taxa (red) and SPF mouse-enriched taxa (green) obtained by LEfSe analysis of 16S sequences. **c** The HOMA mouse-enriched taxa are indicated by a negative LDA score (red), while the taxa enriched by SPF mice have a positive score (green). Taxa at the genus level with different abundances between groups and with an LDA score >3.0 are shown

To further identify the advantages of the HOMA mouse model, we compared the oral microbiota of HOMA mice with SPF mice. PCA revealed that the donor oral microbiota clustered closely with the HOMA mouse but were distinct from SPF mouse microbiota, especially in PC1 (57.91%) (Fig. 2a). The oral microbiota of HOMA mice differed from that of SPF mice in taxonomic structure. Dominant genus-level taxa present in the donor saliva sample were significantly more abundant among HOMA mice than SPF mice, including *Veillonella, Fusobacterium, Streptococcus* and *Haemophilus* (Fig. 2b, c).

### Biogeography of the host gut-selected oral microbiota

**Fig. 3:**
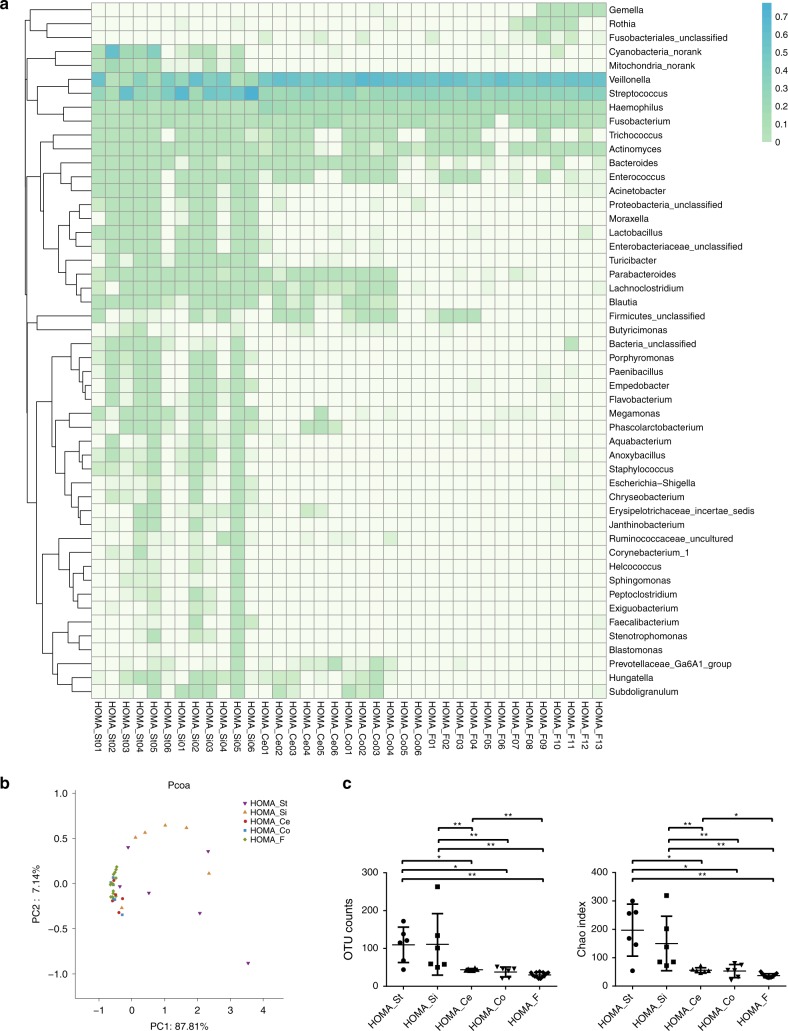
Biogeography of gut-selected oral microbiota. **a** Heatmap of specimens showing the relative abundance of the main identified bacteria at the genus taxonomic level in each segment of HOMA mouse guts, including stomach (St), small intestine (Si), caecum (Ce), colon (Co) and faeces (F). **b** PCoA score plot of the microbiota from each segment of HOMA mouse guts based on unweighted UniFrac metrics. **c** The Kruskal–Wallis test was used to compare the difference between each segment of HOMA mouse guts in the OTU count and Chao index (*P < 0.05, **P < 0.01)

The 16S rRNA gene sequencing survey revealed that the oral bacteria colonised various segments of the gut. In the stomach, eighteen genus-level taxa were detected, with a relative abundance of more than 0.1% on average, eleven of which had a relative abundance exceeding 0.5% on average. In the small intestine, the relative abundances of 23 genus-level taxa exceeded 0.1% on average. Those with a relative abundance greater than 0.5% on average were *Streptococcus, Veillonella, Haemophilus, Enterococcus, Fusobacterium, Acinetobacter, Enterobacteriaceae_unclassified*, and *Bacteroides*. In the caecum, only six genus-level taxa were detected, with a relative abundance greater than 0.1% on average, including *Veillonella, Streptococcus, Haemophilus, Fusobacterium, Bacteroides* and *Trichococcus*. Genus-level taxa with a relative abundance greater than 0.1% in the colon were the same as those in the caecum. The main genus-level taxa in the whole gut were *Streptococcus, Veillonella, Haemophilus, Fusobacterium, Trichococcus* and *Bacteroides* (Fig. 3a, Table S2). All six main genus-level taxa in the gut were also the dominant genus-level taxa (>1%) in the mouth of the HOMA mouse (Table S1). Although the microbial communities colonising various regions shared some main bacteria, the differences among them were clear. Principal coordinates analysis (PCoA) showed that microbial communities present in the caecum, colon, and faeces clustered together and were distinct from those in the stomach and small intestine (Fig. 3b). OTU counts significantly decreased from the stomach and small intestine to the distal gut and from the caecum to faeces, as did the Chao index (Fig. 3c). Distal gut communities were depleted to a low diversity consortium. The relative abundances of *Acinetobacter, Enterobacteriaceae_unclassified, Lactobacillus, Turicibacter, Proteobacteria_unclassified* and *Moraxella* decreased from the stomach and small intestine to the distal gut and faeces. The relative abundances of *Parabacteroides, Lachnoclostridium* and *Blautia* decreased from the caecum and colon to the faeces (Fig. 3a). These results indicated that the oral bacteria were filtered out by the distal gut.

### Ecological invasion by oral microbiota in the gut

**Fig. 4:**
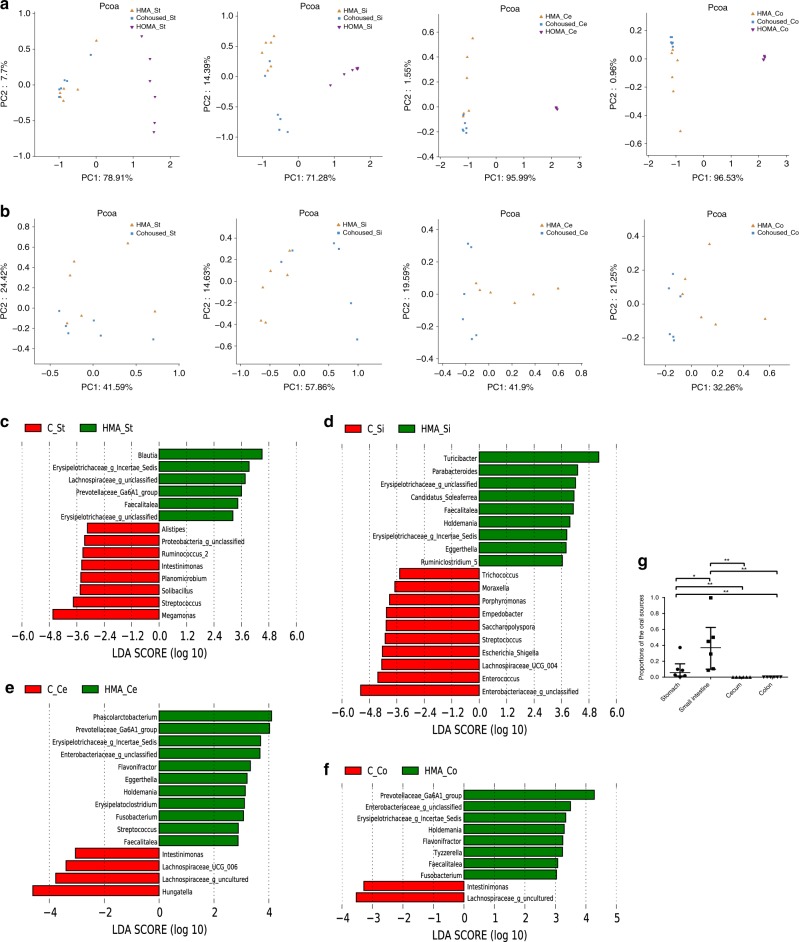
The shift in microbial composition after cohousing. **a** PCoA score plot of the microbiota from each gut segment of HOMA mice, HMA mice and cohoused mice. **b** PCoA score plot of the microbiota from each gut segment from HMA mice and cohoused mice. **c** HMA mouse-enriched genus-level taxa in the stomach are indicated by a positive LDA score (green), while the cohoused (C) mouse-enriched taxa have a negative LDA score (red). **d** The HMA mouse-enriched genus-level taxa in the small intestine are indicated by a positive LDA score (green), while the cohoused mouse-enriched taxa have a negative LDA score (red). **e** The HMA mouse-enriched genus-level taxa in the caecum are indicated by a positive LDA score (green), while the cohoused mouse-enriched taxa have a negative LDA score (red). **f** The HMA mouse-enriched genus-level taxa in the colon are indicated by a positive LDA score (green), while the cohoused mouse-enriched taxa have a negative LDA score (red). **g** Microbial Source Tracker analysis showed the proportions of the different sources present in the microbiota of the cohoused mice in each gut segment. The Kruskal–Wallis test was used to compare the proportions of the oral sources present in each gut segment of the cohoused mice (*P < 0.05, **P < 0.01)

PCoA revealed that the microbial communities in every segment could not be distinguished by the original grouping 28 days after cohousing (Fig. 4a). Therefore, the gut microbiota of the cohoused mice could be regarded as an aggregate, regardless of the original mouse group. The microbial communities of cohoused mice were closely clustered with those of HMA mice and distinct from those of HOMA mice in every segment (Fig. 4a), suggesting that the oral microbiota was unable to challenge the dominant position of the gut microbiota in the gut. Interestingly, further analysis without HOMA mice showed that the microbial communities of cohoused mice could also be separated from HMA mice in every segment (Fig. 4b). These results indicated that although the oral microbiota was almost protected by the gut microbiota barrier, it reshaped the native gut microbiota. To further understand the effect of the oral microbiota on the community composition of the gut microbiota, LEfSe analysis was used. In the stomach, seven genus-level taxa were significantly increased from HMA mice to cohoused mice. One of the seven genus-level taxa was *Streptococcus*, which was the dominant genus (relative abundance > 1%) in the mouth of the HOMA mouse (Fig. 4c). In the small intestine, seven genus-level taxa were significantly increased from HMA mice to cohoused mice, six of which were dominant genera in the mouth of the HOMA mouse: *Enterococcus, Streptococcus, Empedobacter, Porphyromonas, Moraxella* and *Trichococcus* (Fig. 4d). In the distal gut, four genus-level taxa were significantly increased from HMA mice to cohoused mice. but none was the dominant genera in the mouth (Fig. 4e, f). Microbial Source Tracker was used to analyse the effects of cohousing on the flow of microbes between cage mates, which allowed us to determine whether the assembly processes were involved in shaping the communities. The results revealed significant ecological invasion by oral bacteria in the small intestine (Fig. 4g).

### Porphyromonas competed for colonisation with the small intestinal microbiota

**Fig. 5:**
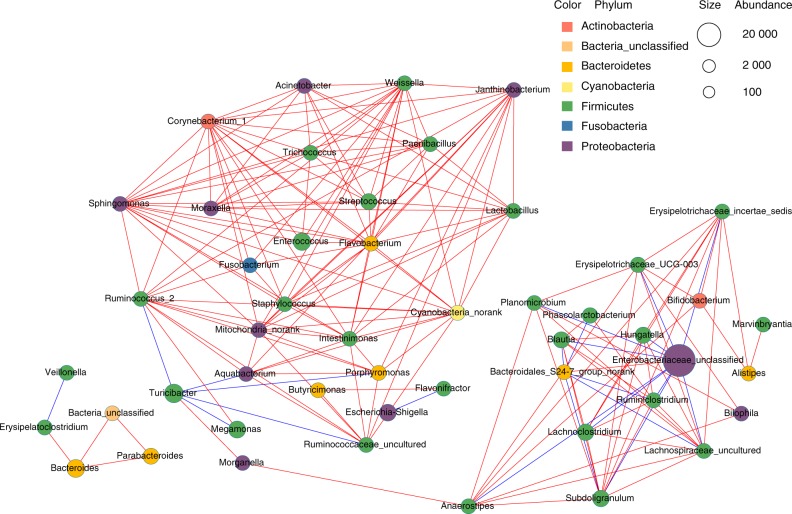
The co-occurrence network was generated from the small intestinal microbiota of the cohoused mice. Different coloured edges represent a positive (red) and a negative (blue) correlation, respectively. Each node represents a genus-level taxon, and the size of each node is proportional to the abundance. The colour of the nodes indicates their classification at the phylum level

To further study the functional positions of oral bacteria in the microbial community colonising the small intestine, the co-occurrence network of the top 50 abundant genus-level taxa was used. *Porphyromonas* was found to correlate negatively with *Turicibacter* (Fig. 5). Before invasion by the oral microbiota, *Turicibacter* was the most dominant genus in the small intestine with the highest relative abundance (40.40% on average). Following invasion by the oral microbiota, the relative abundance of *Porphyromonas* increased significantly, and the abundance of *Turicibacter* decreased to 8.79% on average (Fig. 4d, Fig. S1). Moreover, *Porphyromonas* was found to correlate positively with these genera dominating the mouth of the HOMA mouse, including *Streptococcus, Enterococcus, Acinetobacter, Moraxella, Trichococcus, Fusobacterium, Flavobacterium* and *Lactobacillus* (Fig. 5, Table S1). These results suggested that *Porphyromonas*, as common oral bacteria, had a key role in competing for colonisation with the native main genus in the small intestine.

## Discussion

In the past years, cumulative research data have implied a tight association between dysbiosis of the oral microbiota and diseases.^3,6,7,16,22^ However, it has been difficult to verify the contribution of the oral microbiota to diseases via clinical studies due to their limitations. The lack of understanding of the effect and pathogenic mechanism of dysbiotic oral microbiota manifests in a great gap between the large amount of data and clinical applications.^23^ Thus, for oral microbiota investigations, the establishment of a HOMA mouse model can have an important role in translational medicine, similar to the HMA mouse model. In the present study, 84.78% (39 of 46) of the genus-level taxa were from donor saliva, similar to the HMA mouse model receiving 11 of 12 bacterial classes, and 88% (58 of 66) of the genus-level taxa were human.^12^ Additionally, in subsequent study, we inoculated the contents of another two donor salivary glands into GF mice and obtained similar results.^24^ Additionally, the HOMA mouse was a better representative for the donor than traditional SPF mice (Fig. 2a). Therefore, it is not difficult to conclude that the HOMA mouse model was established successfully. Currently, the HMA mouse is an ideal model to study the role of the disease-associated gut microbiome.^11^ In future, we believe that the HOMA mouse model could be used to investigate the effect of a dysbiotic oral microbiota on oral diseases, such as dental caries, periodontics and oral cancer. In addition to oral disease, the HOMA mouse model will be applied to verify whether the oral microbiota is associated with some digestive systemic diseases.

In most previous studies, the faecal microbiota was collected to represent the gut microbiota; however, some researchers have had different opinions and have suggested to divide the digestive tract into different sections to study the gut microbiota.^25^ By collecting ileostomy samples from humans, Zoetendal et al.^26^ found that the small intestine was enriched with *Streptococcus* sp. and *Escherichia coli*. Interestingly, in the present analysis, invasion by oral bacteria into the small intestine increased the relative abundance of *Streptococcus* and *Enterobacteriaceae* (Fig. 4d). Furthermore, in the small intestines of the cohoused mice, nearly 40% of the taxa were from oral microbial communities, which reshaped the community composition in the small intestine of the HMA mouse (Fig. 4g). Thus, especially in the small intestine, the oral microbiota had an important role in building the integrated gut microbiota.

In the present study, oral bacteria overcame the host physical barrier and colonised the gut in HOMA mice (Fig. 3a, Table S2). However, in cohoused mice, the oral bacteria showed minimal colonisation of the gut, especially the distal gut (Fig. 4g). This result is consistent with a previous study,^20^ in which all the distal guts of HMA mice cohoused with mice with the microbiota from soil or zebrafish were dominated by caecum-derived microbiota at 7 days after cohousing. These results indicated that gut microbiota has an important role as a barrier in resisting the foreign bacteria from mouth. This resistance might due to greater acceptability in the gut of the gut microbiota than the oral microbiota, and the creation of a more stable microenvironment by the gut microbiota to resist foreign bacteria. However, the microbiota barrier of the gut was not consistently indestructible, especially in the small intestine, where six of seven increasing genus-level taxa in the cohoused mice were dominant genera in the mouth of the HOMA mouse, including *Porphyromonas* (Fig. 4d). As a key oral genus to overcome the gut microbiota barrier, *Porphyromonas* was tightly associated with these genera that dominated the mouth of the HOMA mouse, but it correlated negatively with *Turicibacter*, the most dominant genus in the small intestine of HMA mice. Prior to invasion by oral microbiota, the relative abundance of *Turicibacter* in other regions of the gut was lower than that in the small intestine (Fig. S1), which might explain why more oral bacteria invaded the small intestine instead of the other regions. The small intestine is responsible for the majority of substance transformation^27^ and is covered by a thinner mucin layer than the distal gut.^28^ Thus, the small intestinal microbiota more effectively impacts digestive systemic health, suggesting that ecological invasion in the small intestine by *Porphyromonas* had a marked effect on digestive systemic health. For example, oral administration of *Porphyromonas gingivalis*, belonging to *Porphyromonas*, has been confirmed to induce gut microbiota dysbiosis and impair mucosal barrier function, leading to the dissemination of *Enterobacteria* to the liver.^29,30^

Another interesting phenomenon is revealed by the barrier function of the gut microbiota. *Fusobacterium* overcame the physical barrier and became the dominant genus in the gut of the HOMA mouse. However, after receiving the gut microbiota by cohousing, the abundance of *Fusobacterium* decreased markedly, and even the gut microbiota barrier was partly overcome by oral microbiota in the small intestine. *Fusobacterium* was still stopped by the microbiota barrier, but the resistance to *Fusobacterium* was supported by the gut microbiota from a healthy donor here. Those individuals suffering CRC fail to resist *Fusobacterium*.^15,31,32^ The accumulating *Fusobacterium nucleatum* overcome the defective gut microbiota barrier from the CRC patient and further promote tumour development.^33–35^ In conclusion, resistance from various gut microbial communities is a key point to understand the effect of oral microbiota on gut microbiota and digestive systemic health, and it should be investigated in future studies.

Overall, we first established a HOMA mouse model, which copied the oral microbiota of the human donor. Using this animal model, we found that both physical and microbiota barriers filtrated the oral microbiota in the digestive tract. Additionally, the oral microbiota invaded and profiled the gut microbiota, especially in the small intestine. Oral *Porphyromonas* was the key bacterial species competing with the small intestinal microbiota.

## Materials and methods

### Sample collection from humans

The study was authorised by the Ethical Committee of Sichuan University (WCHSIRB-D-2016-070). The saliva was collected using a sterilised tube from an adult donor with natural dentition without periodontitis or active caries and without the use of antibiotics in the previous 3 months. The donor was required not to brush teeth for 24 h and abstain from food/drink intake for 2 h prior to donating saliva. Faeces were collected from the same person using a sterilised sealable plastic bag. A portion of the saliva and faeces were sent to the lab and inoculated into GF mice within 30 min. The rest was stored immediately at –80 °C.

### Animal husbandry

The animal experimentation protocols were approved by the Ethical Committee of Sichuan University (WCHSIRB-D-2016-118) and the Third Military Medical University. Six-week-old GF male Kunming mice were maintained in the Experimental Animal Research Center at the Third Military Medical University. All GF mice were bred in plastic gnotobiotic isolators, where the temperature and humidity were maintained at 20–26 ℃ and 40%–70%, respectively. They were fed a standard diet (GB-T14924.3-2001) sterilised by 60 co gamma radiation. Thirteen-week-old SPF mice were also maintained in the Experimental Animal Research Center. They were fed in the barrier housing facility.

### Establishment of the HOMA and HMA mouse models

To establish the HOMA mouse model, swabs dipped in 200 μL fresh saliva from the male donor were used to seed oral microbiota in the GF mice (*n* = 13) by swabbing without anaesthesia. Swabbing was performed only once. The HMA mouse model was developed as previously described.^36^ The faeces were resuspended in 10 mL sterile potassium phosphate buffer (0.1 mol·L^−1^, pH 7.2). Eight GF mice were inoculated by intragastric gavage with 1 mL human faeces suspension each, and 2-mL aliquots were spread on the fur. HOMA mice and HMA mice were bred in separated plastic gnotobiotic isolators. After 35 days, oral microbial samples were collected from the HOMA mice with swabs. The oral microbial samples from SPF mice were collected in the same way. Faeces of HOMA mice were also collected. Six of thirteen HOMA mice and six of eight HMA mice were subsequently killed randomly, and the contents of the stomach, small intestine, caecum and colon were collected. All the samples were immediately stored at −80 °C.

### Cohousing experiment

Two HOMA mice and two HMA mice was transferred to a new germ-free plastic isolator containing two GF mice (Fig. 1). These six mice were then distributed into two triads, each of which included a HOMA mouse, a HMA mouse and a GF mouse housed in one cage, by which the animals could exchange components of their microbiota. After 28 days, the cohoused mice were killed, and the contents of the stomach, small intestine, caecum and colon were collected. All these samples were immediately stored at −80 °C.

### 16S rRNA gene sequencing

The samples were processed by Shanghai Majorbio Bio-Pharm Technology Co., Ltd (Shanghai, China). Total DNA was extracted, amplified and sequenced according to standard procedures.^37,38^ Briefly, microbial DNA was extracted using the E.Z.N.A.® Soil DNA Kit (Omega Bio-tek, Norcross, GA, U.S.) according to the manufacturer’s protocol. The DNA concentration was assessed using a Nanodrop (Thermo Scientific), and the quality was determined by agarose gel electrophoresis. Bacterial 16S rRNA gene sequences spanning the variable regions V4-V5 were amplified using the primer 515F_907R. The amplicons were then extracted from 2% agarose gels and further purified using the AxyPrep DNA Gel Extraction Kit (Axygen Biosciences, Union City, CA, U.S.) and quantified by QuantiFluor™-ST (Promega, U.S.). Purified amplicons were pooled in equimolar amounts and subjected to paired-end sequencing (2 × 300) on an Illumina MiSeq platform.

### Bioinformatics and statistical analysis

Raw fastq files were demultiplexed and quality-filtered by QIIME (version 1.9.1).^39^ Operational taxonomic units (OTUs) were clustered with a 97% similarity cut-off using UPARSE (version 7.1). The taxonomy of each 16S rRNA gene sequence was analysed using the RDP Classifier (http://rdp.cme.msu.edu/) against the SILVA rRNA database (http://www.arb-silva.de) with a confidence threshold of 70%. After the elimination of interference sequence, alpha diversity estimator calculations were performed using Mothur v.1.30.2. Phylogenetic beta diversity measures, such as unweighted UniFrac distance metrics analysis, was determined using the representative sequences of OTUs for each sample, and PCA and PCoA were conducted according to the distance matrices. LEfSe analysis (linear discriminant analysis [LDA] coupled to effect size measurements) was conducted to calculate bacteria with significant difference in relative abundance between the groups. Using a normalised relative abundance matrix, LEfSe showed taxa with significantly different abundances, and LDA estimated the effect size of the feature.^37,40^ In this study, a *P* value threshold of 0.05 (Wilcoxon rank-sum test) and an effect size threshold of 3 were used for all bacteria discussed. Microbial Source Tracker analysis was performed using the Source Tracker package based on Bayesian inference.^20,41^ The co-occurrence network of the top 50 abundant genus-level taxa was inferred based on the Spearman correlation matrix with a strict *P*-value threshold (*P* *<* 0.05) and a high correlation value (*r* > 0.6) to filter strong correlations. The combined result was exported to Cytoscape V.3.2.1.^37^

The data were subjected to nonparametric Kruskal–Wallis analysis. Differences were considered significant when *P* < 0.05. SPSS21.0 software (SPSS Inc., Chicago, IL, USA) was used for statistical analysis.

### Data availability

The raw reads were deposited into the NCBI Sequence Read Archive (SRA) database (Accession Number: SRP116564).

## Supplementary information

Table S1

Table S2

Fig. S1
