# Supplementary material for: Oral bacteria colonize and compete with gut microbiota in gnotobiotic mice
Source: Int J Oral Sci. 2019 Mar 5;11(1):10. doi: 10.1038/s41368-018-0043-9 (PMC6399334; doi:10.1038/s41368-018-0043-9)
Supplement: Supplementary file 1 — Table S1 [file 41368_2018_43_MOESM1_ESM.docx]

**Table S1**. Oral microbial taxa of the donor and HOMA mice

| Source | Class | Order | Family | Genus |
| --- | --- | --- | --- | --- |
| Donor | \| Bacteroidia^a^ \| \| --- \| \| Betaproteobacteria^a^ \| \| Negativicutes^a^ \| \| Fusobacteriia^a^ \| \| Bacilli^a^ \| \| Clostridia^a^ \| \| Gammaproteobacteria^a^ \| \| Actinobacteria^a^ \| | \| Bacteroidales^a^ \| \| --- \| \| Neisseriales \| \| Selenomonadales^a^ \| \| Fusobacteriales^a^ \| \| Lactobacillales^a^ \| \| Clostridiales^a^ \| \| Pasteurellales^a^ \| \| Actinomycetales \| | \| Prevotellaceae \| \| --- \| \| Neisseriaceae \| \| Veillonellaceae^a^ \| \| Fusobacteriaceae^a^ \| \| Streptococcaceae^a^ \| \| Pasteurellaceae^a^ \| \| Porphyromonadaceae^a^ \| \| Actinomycetaceae \| \| Lachnospiraceae^a^ \| \| Family_XIII \| \| Peptostreptococcaceae \| | Neisseria  Prevotella_7  Veillonella^a^  Alloprevotella  Prevotella  Fusobacterium^a^  Streptococcus^a^  Porphyromonas^a^  Haemophilus^a^  Actinomyces  [Eubacterium]_nodatum_group |
| HOMA mice | \| Bacilli^a^ \| \| --- \| \| Gammaproteobacteria^a^ \| \| Negativicutes^a^ \| \| Bacteroidia^a^ \| \| Flavobacteriia \| \| Clostridia^a^ \| \| Betaproteobacteria^a^ \| \| Fusobacteriia^a^ \| \| Actinobacteria^a^ \| | \| Lactobacillales^a^ \| \| --- \| \| Pseudomonadales \| \| Selenomonadales^a^ \| \| Enterobacteriales \| \| Bacteroidales^a^ \| \| Flavobacteriales \| \| Clostridiales^a^ \| \| Bacillales \| \| Burkholderiales \| \| Pasteurellales^a^ \| \| Fusobacteriales^a^ \| | \| Streptococcaceae^a^ \| \| --- \| \| Enterococcaceae \| \| Moraxellaceae \| \| Veillonellaceae^a^ \| \| Enterobacteriaceae \| \| Flavobacteriaceae \| \| Porphyromonadaceae^a^ \| \| Pasteurellaceae^a^ \| \| Paenibacillaceae \| \| Lachnospiraceae^a^ \| \| Carnobacteriaceae \| \| Fusobacteriaceae^a^ \| \| Bacteroidaceae \| \| Lactobacillaceae \| \| Comamonadaceae \| | Streptococcus^a^  Enterococcus  Acinetobacter  Veillonella^a^  Enterobacteriaceae_unclassified  Moraxella  Haemophilus^a^  Paenibacillus  Trichococcus  Empedobacter  Fusobacterium^a^  Bacteroides  Flavobacterium  Lactobacillus  Porphyromonas^a^ |

Only the taxa with the relative abundance >1% on average were shown.

^a^ The taxa with a relative abundance >1% on average were found in both the donor and HOMA mice.
