# Supplementary material for: Oral bacteria colonize and compete with gut microbiota in gnotobiotic mice
Source: Int J Oral Sci. 2019 Mar 5;11(1):10. doi: 10.1038/s41368-018-0043-9 (PMC6399334; doi:10.1038/s41368-018-0043-9)
Supplement: Supplementary file 2 — Table S2 [file 41368_2018_43_MOESM2_ESM.docx]

**Table S2**. Oral genus-level taxa in various regions of HOMA mice’s gut

|  | Stomach | Small intestine | Cecum | Colon |
| --- | --- | --- | --- | --- |
| Genera | *Streptococcus ^a^*  *Veillonella ^a^*  *Cyanobacteria_norank ^a^*  *Mitochondria_norank ^a^*  *Haemophilus ^a^*  *Fusobacterium ^a^*  *Enterococcus ^a^*  *Trichococcus ^a^*  *Bacteroides ^a^*  *Lactobacillus*  *Acinetobacter*  *Actinomyces*  *Proteobacteria_unclassified*  *Enterobacteriaceae_unclassified*  *Blautia*  *Lachnoclostridium*  *Parabacteroides*  *Bacteria_unclassified* | *Streptococcus ^a^*  *Veillonella ^a^*  *Haemophilus ^a^*  *Enterococcus ^a^*  *Fusobacterium ^a^*  *Acinetobacter ^a^*  *Enterobacteriaceae_unclassified ^a^*  *Bacteroides*  *Lactobacillus*  *Trichococcus*  *Turicibacter*  *Proteobacteria_unclassified*  *Moraxella*  *Lachnoclostridium*  *Parabacteroides*  *Blautia*  *Cyanobacteria_norank*  *Paenibacillus*  *Empedobacter*  *Porphyromonas*  *Flavobacterium*  *Bacteria_unclassified*  *Actinomyces* | *Veillonella ^a^*  *Streptococcus ^a^*  *Haemophilus ^a^*  *Fusobacterium ^a^*  *Bacteroides*  *Trichococcus* | *Veillonella ^a^*  *Streptococcus ^a^*  *Haemophilus ^a^*  *Fusobacterium*  *Trichococcus*  *Bacteroides* |

Only the taxa with the relative abundance >0.1% on average were shown.

^a^ The taxa with a relative abundance >0.5% on average were shown.
