# Supplementary figures and images for: Oral bacteria colonize and compete with gut microbiota in gnotobiotic mice
Source: Int J Oral Sci. 2019 Mar 5;11(1):10. doi: 10.1038/s41368-018-0043-9 (PMC6399334; doi:10.1038/s41368-018-0043-9)

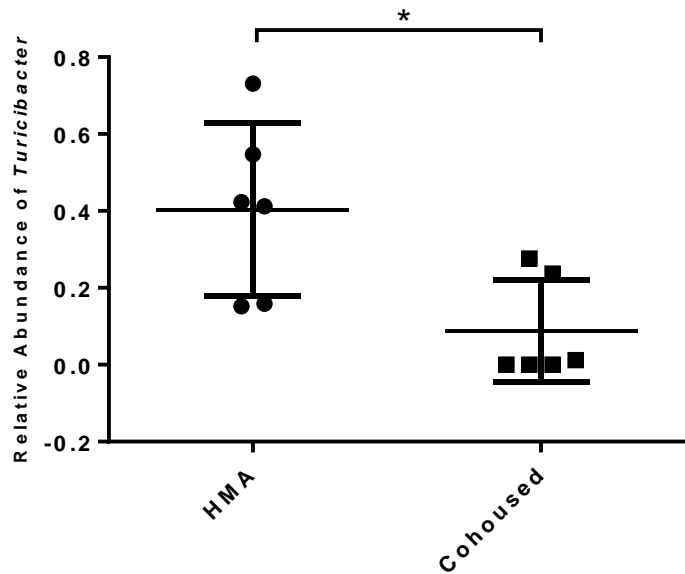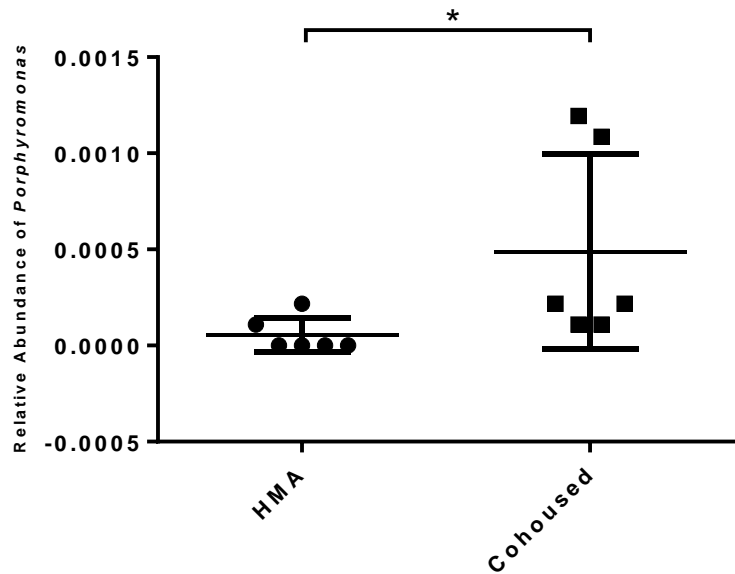

Supplement: Supplementary file 3 — Fig. S1 [file 41368_2018_43_MOESM3_ESM.pdf]
